# 1α,25-Dihydroxyvitamin D3 ameliorates diabetes-induced bone loss by attenuating FoxO1-mediated autophagy

**DOI:** 10.1016/j.jbc.2021.100287

**Published:** 2021-01-13

**Authors:** Yixuan Jiang, Wenqiong Luo, Bin Wang, Zumu Yi, Ping Gong, Yi Xiong

**Affiliations:** 1 State Key Laboratory of Oral Diseases, National Clinical Research Center for Oral Diseases, West China Hospital of Stomatology, Sichuan University, Chengdu, China; 2 Department of Oral Implantology, West China Hospital of Stomatology, Sichuan University, Chengdu, China

**Keywords:** 1α,25-Dihydroxyvitamin D3 (1,25D), autophagy, forkhead transcription factor 1 (FoxO1), osteogenesis, diabetes mellitus, Alp, alkaline phosphatase, BMD, bone mineral density, BV/TV, bone volume per total volume, GFP, green fluorescent protein, Ocn, osteocalcin, Opn, osteopontin, PI3K, phosphoinositide 3-kinase, RAP, rapamycin, RFP, red fluorescent protein, Runx2, runt-related transcription factor 2, T2DM, type 2 diabetes mellitus, Tb.N, the mean trabecular number, Tb.Sp, the mean trabecular separation, TRAP, tartrate-resistant acidic phosphatase

## Abstract

Autophagy is vital for maintaining cellular homeostasis through removing impaired organelles. It has recently been found to play pivotal roles in diabetes mellitus (DM), which is associated with increased bone fracture risk and loss of bone density. However, the mechanism whereby autophagy modulates DM-induced bone loss is not fully elucidated. Previous work has shown that 1α,25-Dihydroxyvitamin D3 (1,25D) exerts positive effects on autophagy, thus affecting bone metabolism. Here, we investigated whether autophagy was involved in the regulation of diabetic bone metabolism. Using Micro-CT, Elisa, histology, and histomorphometry analysis, we demonstrated that 1,25D rescues glucose metabolism dysfunction and ameliorates bone loss in diabetic mice. *In vitro*, 1,25D alleviated primary osteoblast dysfunction and intracellular oxidative stress through reducing prolonged high-glucose-mediated excessive autophagy in primary osteoblasts, reflected by decreased protein level of Beclin1 and LC3. Of note, the autophagy activator rapamycin (RAP) ablated the positive effects of 1,25D in diabetic environment, leading to a marked increase in autolysosomes and autophagosomes, examined by mRFP-GFP-LC3 fluorescence double labeling. The excessive autophagy induced by high glucose was deleterious to proliferation and differentiation of primary osteoblasts. Additionally, biochemical studies identified that PI3K/Akt signaling could be activated by 1,25D, resulting in the inhibition of FoxO1. We confirmed that FoxO1 deficiency alleviated high-glucose-induced autophagy and improved biological functions of primary osteoblasts. Together, our results suggest that the PI3K/Akt/FoxO1 signaling pathway is involved in the osteoprotective effect of 1,25D by attenuating autophagy in diabetes, providing a novel insight for the prevention and treatment of diabetes-caused bone loss.

Diabetes mellitus (DM), a chronic systematic disease, is rapidly growing in the whole world. Apart from some well-known complications of diabetes, the development of DM has also posed great threat to bone health, thus affecting quality of human life (1, 2). Studies have revealed that both type 1 and type 2 diabetes are responsible for impaired bone turnover and remodeling, with enhanced bone metabolism disorders and even worse fracture outcomes (3, 4). 1α,25-Dihydroxyvitamin D3 (1,25D) is the active form of vitamin D. The potential of 1,25D supplementation in the treatment of many disorders, for example, cancer, kidney diseases, has been well documented (5, 6). There is growing literature demonstrating that vitamin D could be a drug candidate for DM since it has protective impacts on β-cell function, inflammatory response, and insulin secretion (7, 8). Also, the role of 1,25D in the osteogenesis under diabetic condition has generated substantial interest for its clear effects on bone metabolism. However, the underlying function of 1,25D and molecular signal transduction pathways involved remains largely unclear.

Autophagy is a vital mechanism sustaining cellular homeostasis, which is involved in activating stress responses, removing impaired organelles, eliminating misfolded proteins, regulating growth, and renewing cellular development (9). Dysregulation of autophagy is critical in various diseases, especially cancer, neurodegenerative and inflammatory disorders (10, 11, 12). In addition, the potential relationship between autophagy and diabetes has been increasingly reported. It appears that autophagy might exert positive effects on maintaining insulin homeostasis due to its role in the regulation of β-cell differentiation and survival (13). But research about the role of autophagy in nontargeted cells in diabetes, for example, osteoblastic cells, is limited, which encourages us to investigate the influences of autophagy on diabetic bone metabolism. Furthermore, whether 1,25D regulation in the osteoprotective process is related with autophagy in a high-glucose (HG) environment has not yet been fully established.

Previous research has indicated that 1,25D could activate PI3K/Akt signaling transduction pathway to modulate cellular functions (14, 15). Phosphoinositide 3-kinase (PI3K), as a kind of lipid kinases, plays a central role in many signal pathways and then affects cellular biology (16). Akt could be activated by PI3K *via* phosphorylation. Malfunction of PI3K/Akt signaling is partially required for the insulin resistance and oxidative stress, thus accelerating the progression of diabetes (17, 18). Forkhead box protein O1 (FoxO1) is a key transcription factor widely expressed in the bone tissues. In osteoblasts, it could mediate downstream genes participated in antioxidant response, differentiation, viability, and apoptosis (19, 20, 21). Importantly, FoxO1 has been identified as one of the potential targets of Akt, which could be inhibited *via* phosphorylation process (22, 23). Studies have suggested that suppression of FoxO1 improves the ability of insulin to maintain glucose levels. While insulin could promote the formation of osteoblasts and further inhibit the FoxO1 activity, highlighting the potential function of FoxO1 in coordinating bone turnover with glucose homeostasis (24, 25). Besides, FoxO1 is regarded as a crucial mediator contributing to the modulation of autophagy in various cells, such as cardiomyocytes, chondrocytes, and adipocytes, thereby affecting multiple physiological and pathological processes (26, 27, 28). Therefore, FoxO1 might also play an important role in regulating autophagic activity in osteoblasts and then influencing osteogenic functions.

Based on this evidence, this study aimed to explore the effects of 1,25D on autophagy in osteogenesis *in vivo* and *vitro* under diabetic situation and determine the role of FoxO1 in this process. We revealed herein that 1,25D could attenuate bone loss through alleviating diabetes-mediated excessive autophagy *via* PI3K/Akt/FoxO1 signaling pathway. In other words, 1,25D inhibited hyperglycemia-induced autophagy through FoxO1 inactivation, thereby enhancing differentiation and proliferation in HG-treated osteoblasts. Besides, overexpression of FoxO1 enhanced autophagic activity and disrupted the osteoprotective effects of 1,25D, but deletion of FoxO1 reversed these adverse influences on osteoblasts functions. Our results suggested novel perspectives of 1,25D treatment in maintaining normal bone metabolism during diabetes therapy.

## Results

### 1,25D ameliorates diabetes-induced bone loss in diabetic mice

**Figure 1:**
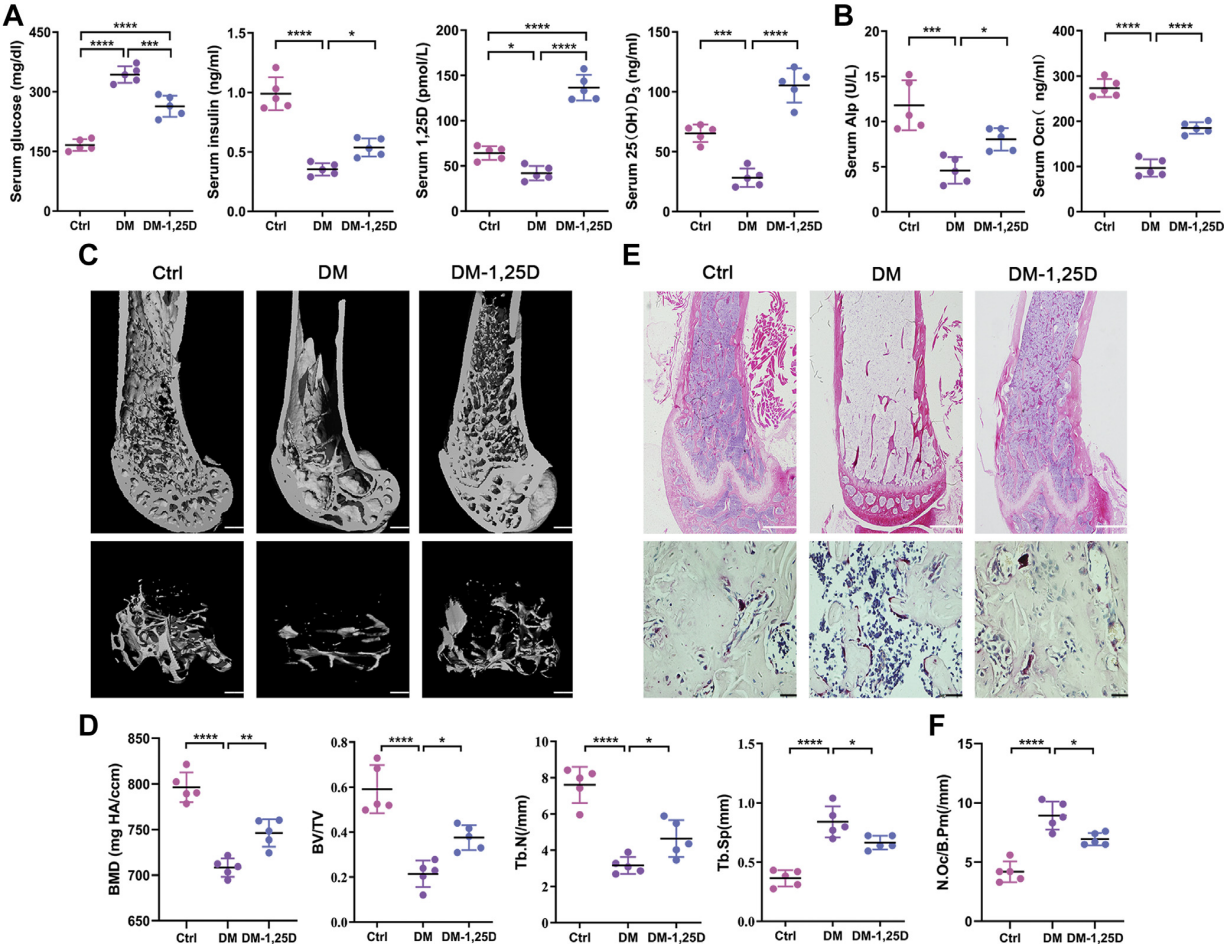
**1,25D ameliorates diabetes-induced bone loss *in vivo*.** Serum level of glucose, insulin, 1,25D, 25(OH)D_3_ (*A*), and alkaline phosphatase (Alp), osteocalcin (Ocn) (*B*) in diabetic mice with or without 1,25D treatment tested by ELISA assay, n = 5. *C*, the representative micro-CT 3D reconstruction images of distal femurs, scale bar = 200 μm. *D*, quantitative analysis of bone morphology including bone mineral density (BMD), bone volume per total volume (BV/TV), mean trabecular number (Tb.N), and mean trabecular separation (Tb.Sp) in femoral bone from different groups, n = 5. *E*, HE of sagittal sections from distal femurs, n = 5. Scale bar = 200 μm. And TRAP staining, n = 5. Scale bar = 20 μm. *F*, quantitativosteoclasts number in distal femur bone, n = 5. Data are expressed as mean ± standard deviation (SD) of three independent experiments, ∗*p* < 0.05, ∗∗*p* < 0.01, ∗∗∗*p* < 0.001, ∗∗∗∗*p* < 0.0001. Ctrl, control; DM, diabetes mellitus.

*In vivo*, wild-type (WT) mice were used. Firstly, we found that serum glucose level significantly increased and insulin decreased in DM group compared with the control group (Fig. 1*A*). To determine the level of our therapeutic 1,25D dose regime in mice, serum 1,25D concentration was tested 4 h after three consecutive times of administration. Results showed that serum level of 1,25D increased sharply in diabetic mice treated with 1,25D. And 1,25D reversed those negative effects caused by hyperglycemia in DM group and greatly enhanced the circulating 25(OH)D_3_ concentration (Fig. 1*A*). Meanwhile, we found that serum value of osteocalcin (Ocn) and alkaline phosphatase (Alp), the biomarkers of osteogenesis, were inhibited dramatically in DM group. But increased levels of Ocn and Alp were detected in DM-1,25D group, with statistical significances in contrast to DM group (Fig. 1*B*).

To observe the effects of 1,25D on bone tissues, micro-CT analysis was applied to determine details of bone mass and microstructure. The 3D images from Micro-CT measurements displayed obvious bone loss of femurs in DM group (Fig. 1*C*). Compared with control group, diabetic mice showed a sharp decline in bone mineral density (BMD), bone volume per total volume (BV/TV), and mean trabecular number (Tb.N), yet a significant rise in mean trabecular separation (Tb.Sp), implying loss of bone mass and structural deterioration in DM group. Nevertheless, 1,25D treatment ameliorated changes in these parameters caused by diabetes, displaying increased BMD, BV/TV, Tb.N and decreased Tb.Sp (Fig. 1*D*). Also, HE and tartrate-resistant acid phosphatase (TRAP) staining revealed that 1,25D rescued diabetes-induced bone loss in mice (Fig. 1*E*), with a remarkably reduced osteoclasts number induced by diabetes (Fig. 1*F*). Additionally, there were no statistical difference between Ctrl group and Ctrl-1,25D group in terms of bone trabecular parameters and serum values of markers tested above (Tables S1 and S2). These results indicated that 1,25D could ameliorate bone loss in diabetic mice.

### 1,25D improves osteogenesis dysfunction caused by hyperglycemia *in vitro* and *in vivo*

**Figure 2:**
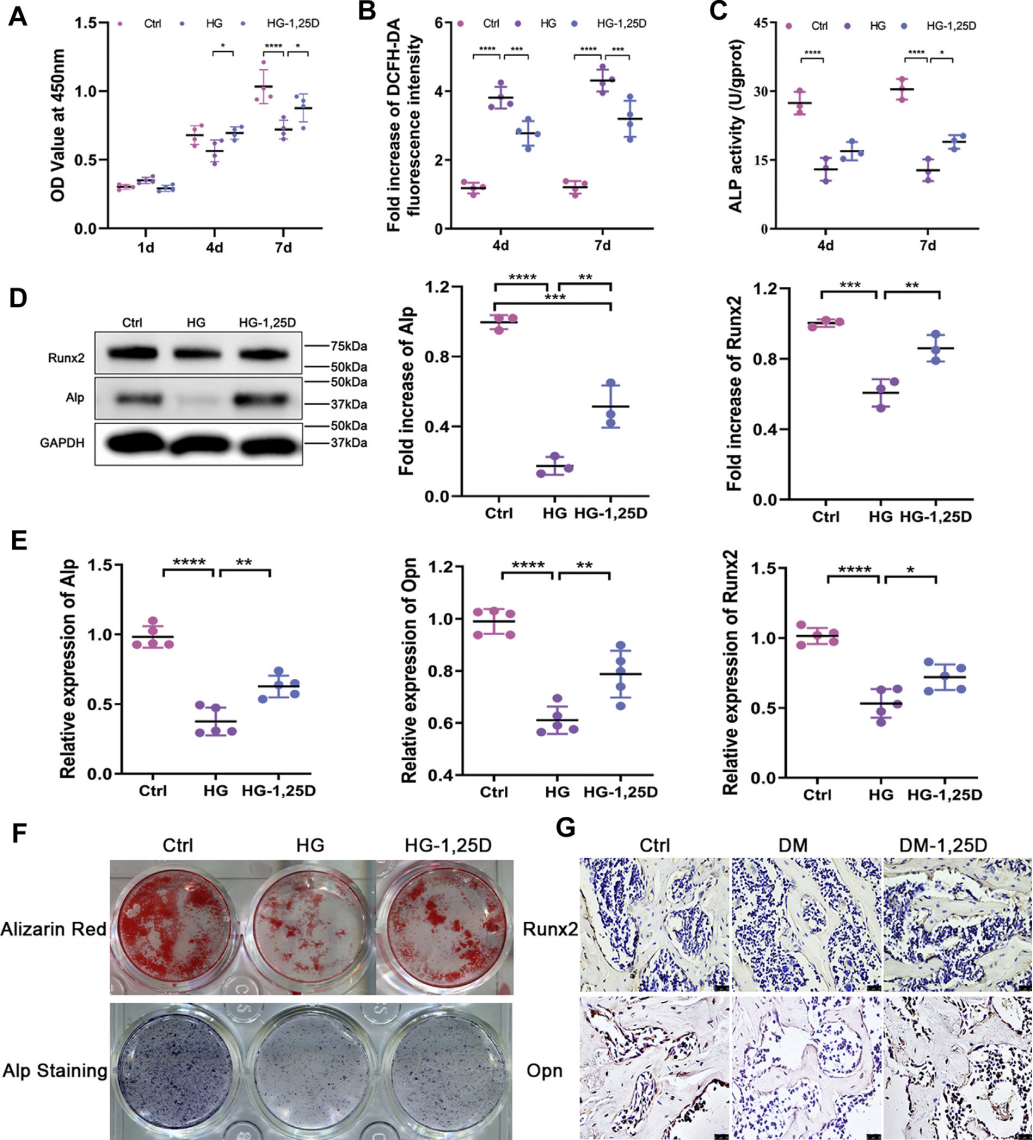
**1,25D promotes osteogenic differentiation *in vitro* and *in vivo*.***A*, cell viability determined by CCK-8 assay at 1 day, 4 days, and 7 days in osteoblasts cultured in different conditions, n = 4. *B*, intracellular reactive oxygen species (ROS) level at 4 days and 7 days in different groups, n = 4. *C*, osteogenic differentiation assessed by alkaline phosphatase (ALP) activity at 4 days and 7 days, n = 3. *D*, quantification of protein levels of Alp and runt-related transcription factor 2 (Runx2) examined by western blot, with glyceraldehyde-3-phosphate dehydrogenase (GAPDH) as loading control, n = 3. *E*, the mRNA expressions of Alp, osteopontin (Opn), and Runx2 tested using real time-qPCR, n = 5. Data were normalized to GAPDH. *F*, alizarin red and Alp staining of osteoblasts after 21-day osteogenic induction in different groups. *G*, immunohistochemical staining of Runx2 and Opn in the femurs from diabetic mice with or without 1,25D treatment, n = 4, scale bar = 25 μm. Data obtained in three independent experiments are presented as mean ± standard deviation (SD), ∗*p* < 0.05, ∗∗*p* < 0.01, ∗∗∗*p* < 0.001, ∗∗∗∗*p* < 0.0001. Ctrl, control; DM, diabetes mellitus; HG, high glucose.

To explore the impacts of 1,25D on osteogenesis over diabetic hyperglycemia condition, primary osteoblasts were exposed to high concentration of glucose. CCK-8 assay is a rapid and highly sensitive test widely used to study cell proliferation. Number of viable cells was detected based on WST tetrazolium salt following the manufacturer’s instructions (CCK-8, Dojindo) (29). As shown in Figure 2*A*, CCK-8 test revealed that the proliferative activity of primary osteoblasts slightly increased when cells were cultured in high glucose at 1 day, but it was gradually suppressed as the incubation period prolonged, with a significant difference compared with control group at 7 days. However, 1,25D treatment rescued cell viability at 4 days and 7 days. Intracellular reactive oxygen species (ROS) level, which has been identified as a reflection of oxidative stress level in cells, displayed a prominent increase in HG group. But 1,25D downregulated ROS level markedly in HG-1,25D group, indicating its resistance to cellular redox imbalance caused by elevated glucose (Fig. 2*B*).

ALP activity, Alp staining, and Alizarin Red staining were conducted to evaluate the differentiation potential of primary osteoblasts in HG environment. Results in Figure 2, *C* and *F* showed impaired osteogenic differentiation following HG treatment. Nevertheless, 1,25D attenuated this adverse impact on primary osteoblasts differentiation, showing higher ALP activity in HG-1,25D group than HG group. Also, exposure to high concentration of glucose led to fewer calcium nodules in medium after 21-day osteogenic induction, yet 1,25D treatment promoted the alizarin red-positive nodules, restoring the capacity of late osteogenic differentiation for primary osteoblasts (Fig. 2*F*). Besides, protein levels of Alp and runt-related transcription factor 2 (Runx2) decreased drastically in HG group, while 1,25D treatment led to enhanced expression levels of these markers (Fig. 2*D*). In accordance with the data above, the mRNA expressions of Alp, osteopontin (Opn), and Runx2 showed similar variations (Fig. 2*E*). *In vivo*, it was also found that Runx2 and Opn expressions exhibited consistent results as observed *in vitro* according to immunohistochemistry (Fig. 2*G*). These findings suggested that 1,25D could promote osteogenesis under diabetic hyperglycemia situations *in vitro* and *in vivo*.

### 1,25D alleviates high-glucose-mediated autophagy in primary osteoblasts

**Figure 3:**
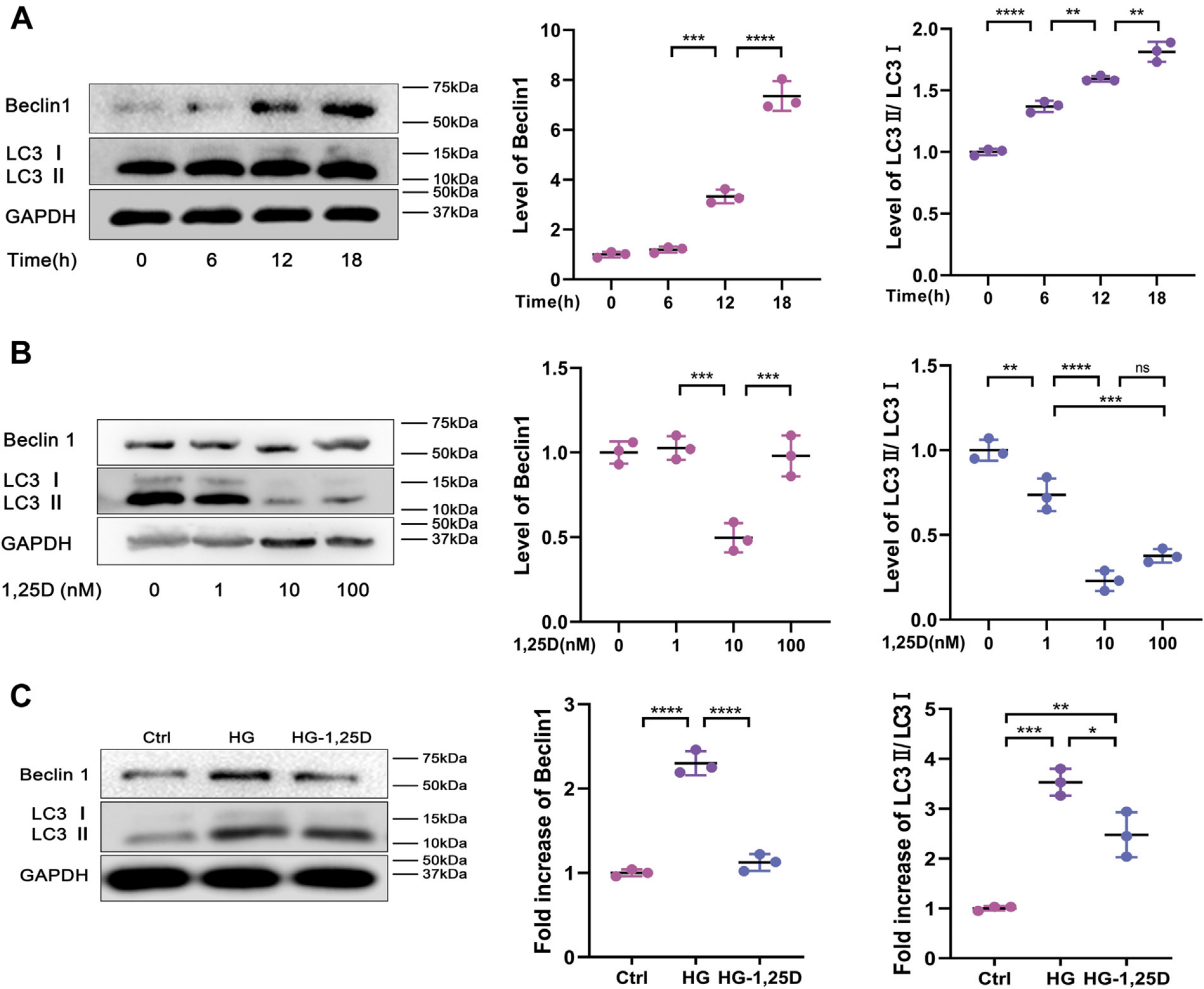
**Effects of 1,25D on autophagy in osteoblasts exposed to high glucose.***A*, protein expression levels of Beclin1, LC3II/LC3I in osteoblasts tested by western blot analysis at 0, 6, 12, 18 h after high glucose intervention, n = 3. *B*, protein levels of Beclin1 and LC3II/LC3I detected by western blot analysis in high glucose (HG)-treated osteoblasts with the treatment of 1,25D at 0, 1, 10, 100 nM, for selecting optimum dose of 1,25D, n = 3. *C*, changes of protein expression levels of Beclin1, LC3II/LC3I were examined using western blot analysis in different groups, n = 3. Data are expressed as mean ± standard deviation (SD) of three independent experiments, ns represents *p* > 0.05, ∗*p* < 0.05, ∗∗*p* < 0.01, ∗∗∗*p* < 0.001, ∗∗∗∗*p* < 0.0001. Ctrl, control; HG, high glucose.

To investigate the effect of 1,25D on autophagy in primary osteoblasts, western blot analysis was performed to measure the protein expressions of Beclin1 and LC3II/LC3I. Beclin1, one of the most widely studied autophagy-related proteins, participates in many stages of autophagy including autophagosome formation, phagosome maturation, *etc* (30, 31). As another mammalian autophagosome marker protein monitoring the autophagy activity, LC3 could transform into autophagosome-associated lipidated form (LC3II) from cytosolic nonlipidated form (LC3I) when autophagy was stimulated (32). First of all, we observed that Beclin1 expression and LC3II/LC3I ratio in primary osteoblasts were time-dependent following HG treatment. The longer HG treatment it took, the higher levels of Beclin1 and LC3II/LC3I were, suggesting enhanced autophagic activity caused by glycometabolism abnormality in primary osteoblasts (Fig. 3*A*). 1,25D treatment was then conducted in concentration gradient under HG condition. As displayed in Figure 3*B*, 1,25D downregulated Beclin1 expression and LC3II accumulation in a dose-dependent manner. That is, we found that 10 nM 1,25D was most effective and used for subsequent experiments. Then augmentation of Beclin1 and higher ratio of LC3II/LC3I were observed in HG group, but 1,25D supplementation reversed these negative effects notably, reflected by decreased Beclin1 expression and LC3II/LC3I ratio in HG-1,25D group (Fig. 3*C*).

### Osteogenesis is upregulated in high glucose by reduction of autophagy through 1.25D treatment

**Figure 4:**
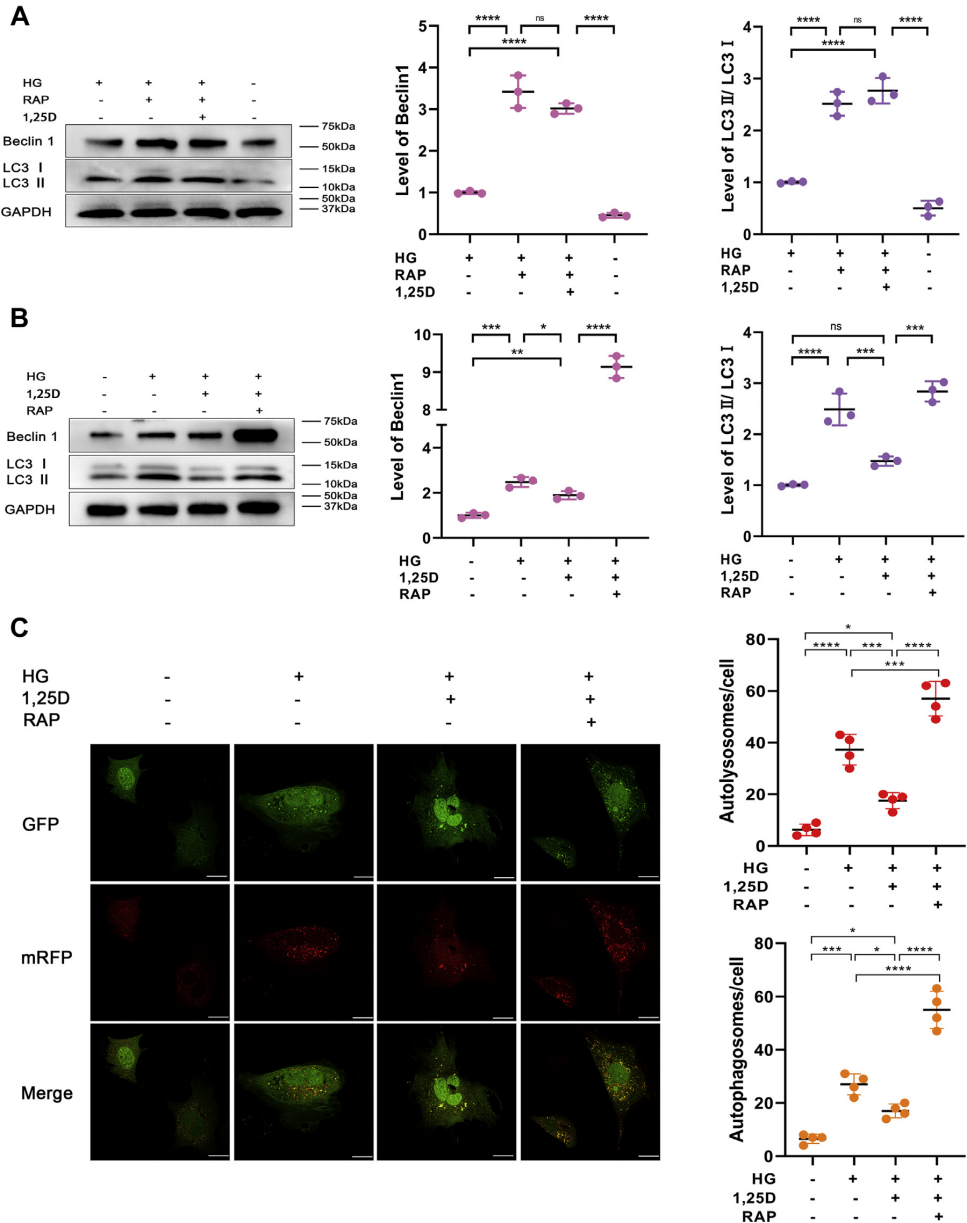
**Effects of rapamycin (RAP) on osteoblasts autophagy in high-glucose environment.** Protein expression levels of Beclin1, LC3 (*A* and *B*) were tested by western blot analysis, n = 3. *C*, representative images and quantification analysis of mRFP-GFP-LC3 fluorescence double labeling, each image is representative of four specimens, scale bar=5 μm. *Red* indicates the number of autolysosomes in osteoblasts exposed to different treatment and *yellow* refers to the number of autophagosomes in cells, n = 4. Data are expressed as mean ± standard deviation (SD) of three independent experiments, ns represents *p* > 0.05, ∗*p* < 0.05, ∗∗*p* < 0.01, ∗∗∗*p* < 0.001, ∗∗∗∗*p* < 0.0001. Cells were incubated with or without 100 nM RAP, HG, high glucose.

**Figure 5:**
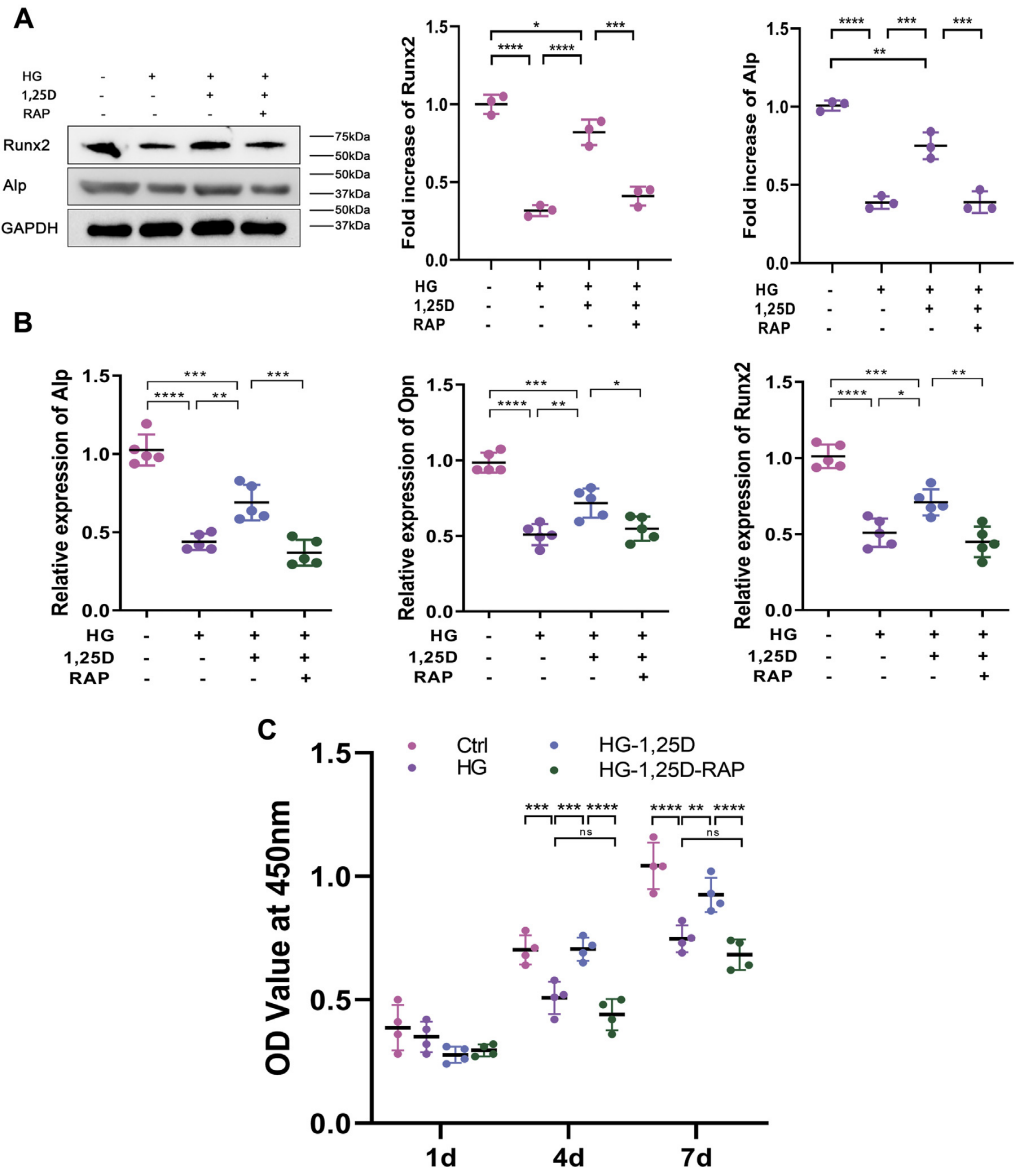
**Effects of excessive autophagy on osteogenic differentiation in high-glucose environment.***A*, protein expression levels of runt-related transcription factor 2 (Runx2) and alkaline phosphatase (Alp) were tested by western blot analysis, n = 3. *B*, relative expressions of Alp, osteopontin (Opn), and Runx2 were calculated by real-time qPCR analysis, with housekeeping gene GAPDH as loading control, n = 5. *C*, cell proliferation determined by CCK-8 assay at 1 day, 4 days, and 7 days in different groups, n = 4. Data are expressed as mean ± standard deviation (SD) of three independent experiments, ns represents *p* > 0.05, ∗*p* < 0.05, ∗∗*p* < 0.01, ∗∗∗*p* < 0.001, ∗∗∗∗*p* < 0.0001. Cells were incubated with or without 100 nM rapamycin (RAP), Ctrl, control; HG, high glucose.

To further examine the role of autophagy in osteogenic function of primary osteoblasts exposed to HG, rapamycin (RAP), as the major agonist for autophagy, was used in this study. Cells were incubated with 100 nM RAP (Sigma). The level of Beclin1 and LC3II/LC3I ratio was enhanced dramatically when RAP was applied alone in primary osteoblasts cultured in HG environment. Although 1,25D could alleviate HG-mediated autophagic activity as the results described above, 1,25D treatment couldn’t mitigate elevated autophagy induced by RAP (Fig. 4, *A* and *B*). To better understand the underlying mechanism, we applied mRFP-GFP-LC3 fluorescence double labeling to detect autophagic flux. Green fluorescent protein, known as GFP, was sensitive to acidic conditions of the lysosome lumen. Red fluorescent protein, in brief, RFP, was used to track autophagy-related protein LC3. The colocalization of green and red fluorescence displayed yellow puncta, which referred to autophagosomes. After lysosomes and autophagosomes fused and autolysosome formed, GFP would weaken even quench in this acidic microenvironment. Thus, only red fluorescence could be detected at this time and red spots indicated autolysosome. The increased number of yellow and red puncta reflected the enhancement of autophagic flux (33). As shown in Figure 4*C*, 1,25D treatment reduced HG-mediated autophagic flux in primary osteoblasts, but this effect was counteracted by RAP, which dramatically increased red and yellow puncta, indicating a higher level of autophagic flux. Simultaneously, protein levels of Runx2 and Alp were inhibited when cells were exposed to HG, but improved under the treatment of 1,25D (Fig. 5*A*). However, the protein expression of these two osteogenic biomarkers decreased significantly when RAP was applied (Fig. 5*A*), accompanied by similar variations of mRNA expressions of Alp, Opn, and Runx2 (Fig. 5*B*). HG and RAP could both lead to a marked decrease in these mRNA expressions compared with the HG-1,25D group (Fig. 5*B*). For cell viability, CCK-8 assay revealed that 1,25D did not always exert positive effects at all time points. The significant differences were observed as time prolonged, especially at 4 days and 7 days, showing the protective role of 1,25D in HG-treated primary osteoblasts. However, RAP exacerbated inhibitory effects on the viability of primary osteoblasts (Fig. 5*C*). Based on these data, we speculated that 1,25D could attenuate HG-induced autophagy and thereby potentiate viability and osteogenic differentiation of primary osteoblasts. Also, we confirmed that excessive autophagy by RAP was detrimental to biological characteristics of primary osteoblasts.

### PI3K/Akt/FoxO1 signaling pathway is involved in the osteoprotective effect of 1,25D by attenuating autophagy in high-glucose environment

**Figure 6:**
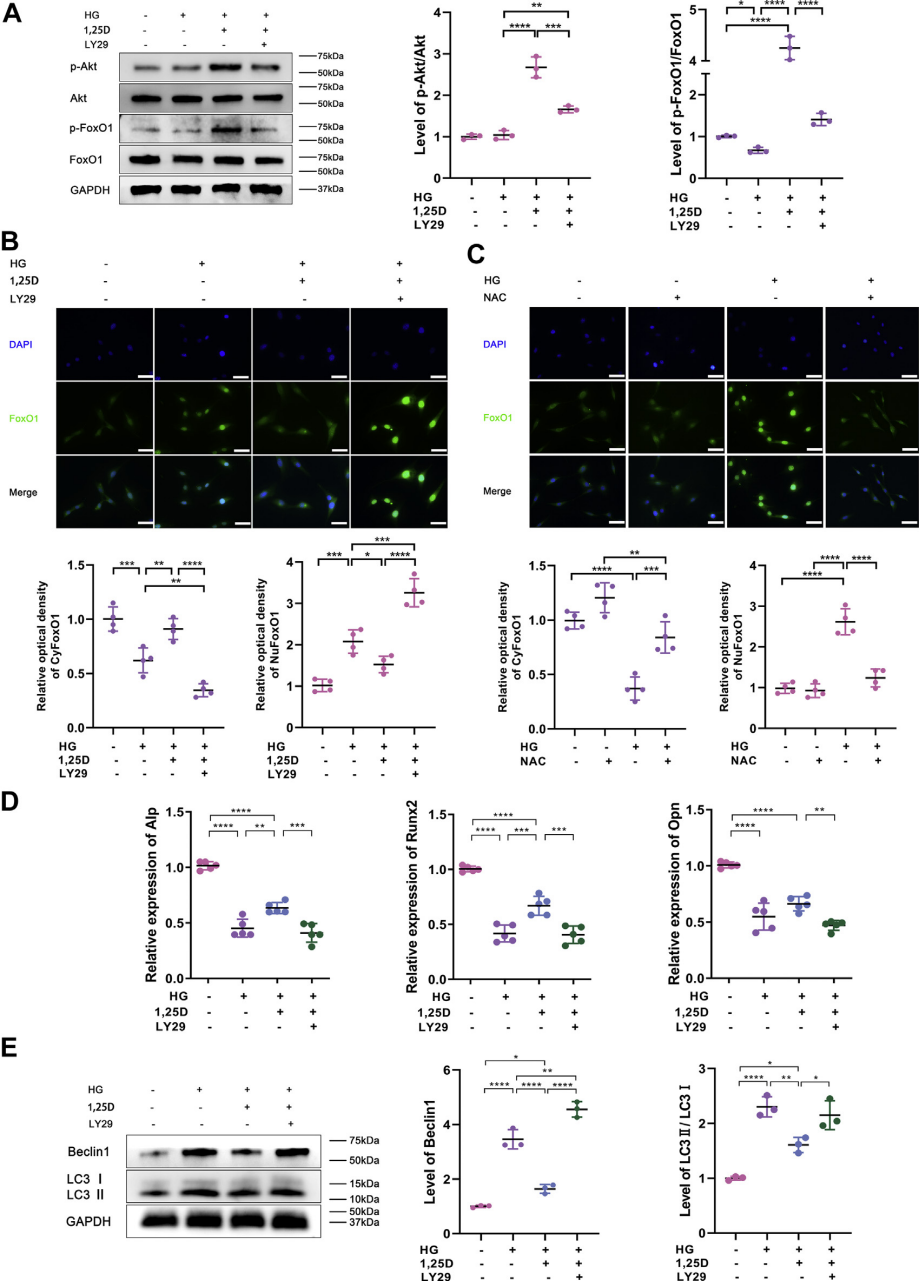
**PI3K/Akt/FoxO1 signaling is involved in the osteoprotective effect of 1,25D.***A*, protein levels of p-Akt, Akt, p-FoxO1, and FoxO1 were detected using western blot, n = 3. *B* and *C*, FoxO1 expression was evaluated by immunofluorescent staining, each image is representative of four specimens. Scale bar = 20 μm. And quantitation of the distribution of FoxO1 between the nucleus and the cytosol was detected by Image Pro Plus software. *D*, relative expressions of alkaline phosphatase (Alp), runt-related transcription factor 2 (Runx2), and osteopontin (Opn) tested by real-time qPCR analysis, n = 5. *E*, protein levels of Beclin1 and LC3II/LC3I were detected using western blot assay, n = 3. Values expressed as mean ± standard deviation (SD) of three independent experiments, ∗*p* < 0.05, ∗∗*p* < 0.01, ∗∗∗*p* < 0.001, ∗∗∗∗*p* < 0.0001. For (*A*, *B*, *D* and *E*), cells were pretreated with or without a 2-h incubation of 1,25D or a 2-h culture of PI3K inhibitor LY294002 (LY29, 50 mol/L). For (*C*), cells were pretreated with or without a 2-h incubation of N-acetylcysteine (NAC), a kind of scavenger of reactive oxygen species (ROS). HG, high glucose.

Previous studies have revealed that PI3K/Akt signaling pathway is critical in many intracellular physiological processes (17, 34). To determine whether PI3K/Akt signaling participates in the effect of 1,25D on the biological function of primary osteoblasts in HG environment, cells were pretreated in a 2-h incubation of PI3K inhibitor LY294002 (LY29, 50 mol/l, Sigma). Given that 1,25D could induce a time-dependent Akt and FoxO1 phosphorylation as previously reported (35), relevant analysis was detected after a 2-h treatment of 1,25D. It was observed that p-Akt/Akt level increased when 1,25D was added in primary osteoblasts cultured in HG (p-Akt is the active form of Akt *via* phosphorylation), which implied that PI3K/Akt signaling was activated by 1,25D. However, p-Akt/Akt ratio declined measurably after LY29 was added. Meanwhile, the production of p-FoxO1, inactive form of FoxO1 *via* phosphorylation, was promoted appreciably in primary osteoblasts exposed to HG with 1,25D treatment. Then the ratio of p-FoxO1/FoxO1 increased, indicating that FoxO1 was restrained by 1,25D treatment, contrary to the results added with LY29 (Fig. 6*A*). Additionally, immunofluorescence assay showed that FoxO1 expression was enhanced and accumulated in nucleus in HG environment, while 1,25D inhibited FoxO1 activity and promoted its nuclear exclusion. The application of LY29 further increased the expression level of FoxO1, promoting the translocation of FoxO1 from cytoplasm to nucleus (Fig. 6*B*). Hyperglycemia-induced oxidative stress was reflected by the increased level of ROS. To further explore the role of ROS in the FoxO1 distribution under HG condition, primary osteoblasts were treated in a 2-h incubation of N-acetylcysteine (NAC, Sigma), a kind of known scavenger of ROS. And we detected FoxO1 distribution between the nucleus and the cytosol after 24 h. Consistent with the data above, HG led to the remarkably increased accumulation of FoxO1 in nucleus. When NAC was added, the shuttle of FoxO1 from nucleus to cytoplasm was promoted, suggesting that FoxO1 activity was suppressed when ROS production decreased (Fig. 6*C*). According to Figure 6*D*, results of real-time qPCR displayed the inhibitory effects of HG and LY29 on the mRNA levels of Alp, Opn, and Runx2, while 1,25D upregulated the expressions of these osteogenic markers and had a positive influence on osteogenic differentiation. And we found that autophagic activity in primary osteoblasts was repressed with 1,25D supplementation, yet enhanced by LY29 under HG condition, showing a higher level of Beclin1 and LC3II/LC3I ratio compared with other groups (Fig. 6*E*). These evidences indicated that PI3K/Akt/FoxO1 signaling pathway was involved in the osteoprotective effect of 1,25D treatment under HG condition, and oxidative stress might play a critical role in this process.

**Figure 7:**
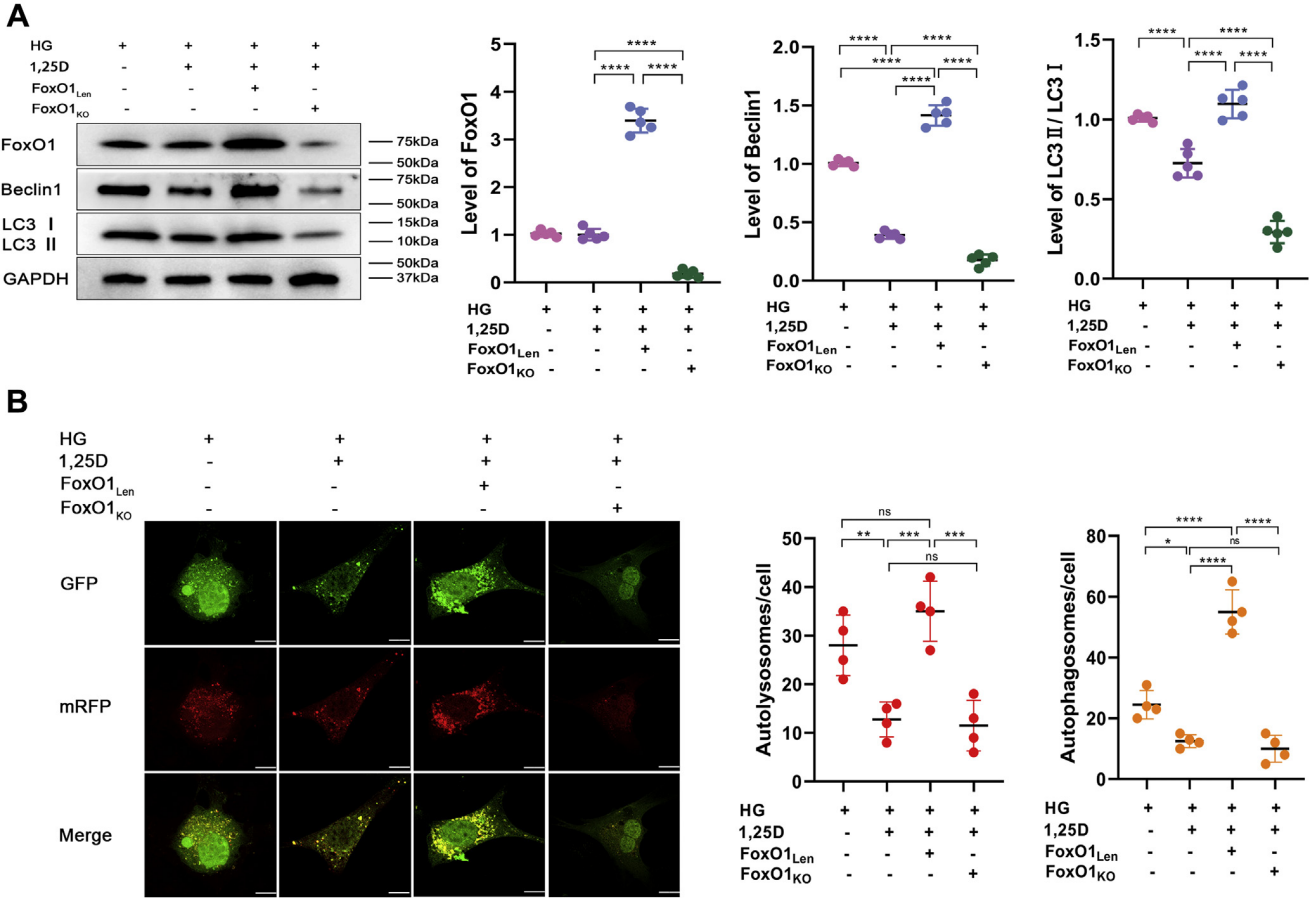
**1,25D and FoxO1 deletion ameliorates osteoblasts autophagy in high-glucose environment.***A*, variations of protein levels of FoxO1, Beclin1 and LC3II/LC3I assessed by western blot in different groups, n = 5. *B*, representative images and quantification analysis of mRFP-GFP-LC3 fluorescence double labeling, scale bar=5 μm, each image is representative of four specimens. *Red* indicates the number of autolysosomes in osteoblasts exposed to different treatment, and *yellow* refers to the number of autophagosomes in cells. Data are expressed as mean ± standard deviation (SD) obtained in 3 ihreendependent experiments, ns represents *p* > 0.05, ∗*p* < 0.05, ∗∗*p* < 0.01, ∗∗∗*p* < 0.001, ∗∗∗∗*p* < 0.0001. FoxO1_KO_, conditional knockout of FoxO1; FoxO1_Len_, lentivirus-mediated overexpression of FoxO1; HG, high glucose.

To further investigate the function and mechanism of FoxO1 in the regulation of diabetic bone metabolism, lentivirus-mediated overexpression of FoxO1 (FoxO1_Len_) in primary osteoblasts was constructed. Also, conditional FoxO1 knockout primary osteoblasts (FoxO1_KO_) were isolated from KO mice and cultured. FoxO1 overexpression led to a remarkable increase of Beclin1 and LC3II/LC3I ratio in HG-treated primary osteoblasts following 1,25D treatment, while FoxO1 knockout exhibited opposite effects on these two autophagic markers (Fig. 7*A*). According to mRFP-GFP-LC3 fluorescence double labeling analysis shown in Figure 7*B*, both 1,25D treatment and FoxO1 knockout could reduce HG-mediated enhanced autophagic flux, while FoxO1 overexpression led to a higher level of intracellular autophagic flux, showing more autolysosomes and autophagosomes in HG-treated primary osteoblasts.

**Figure 8:**
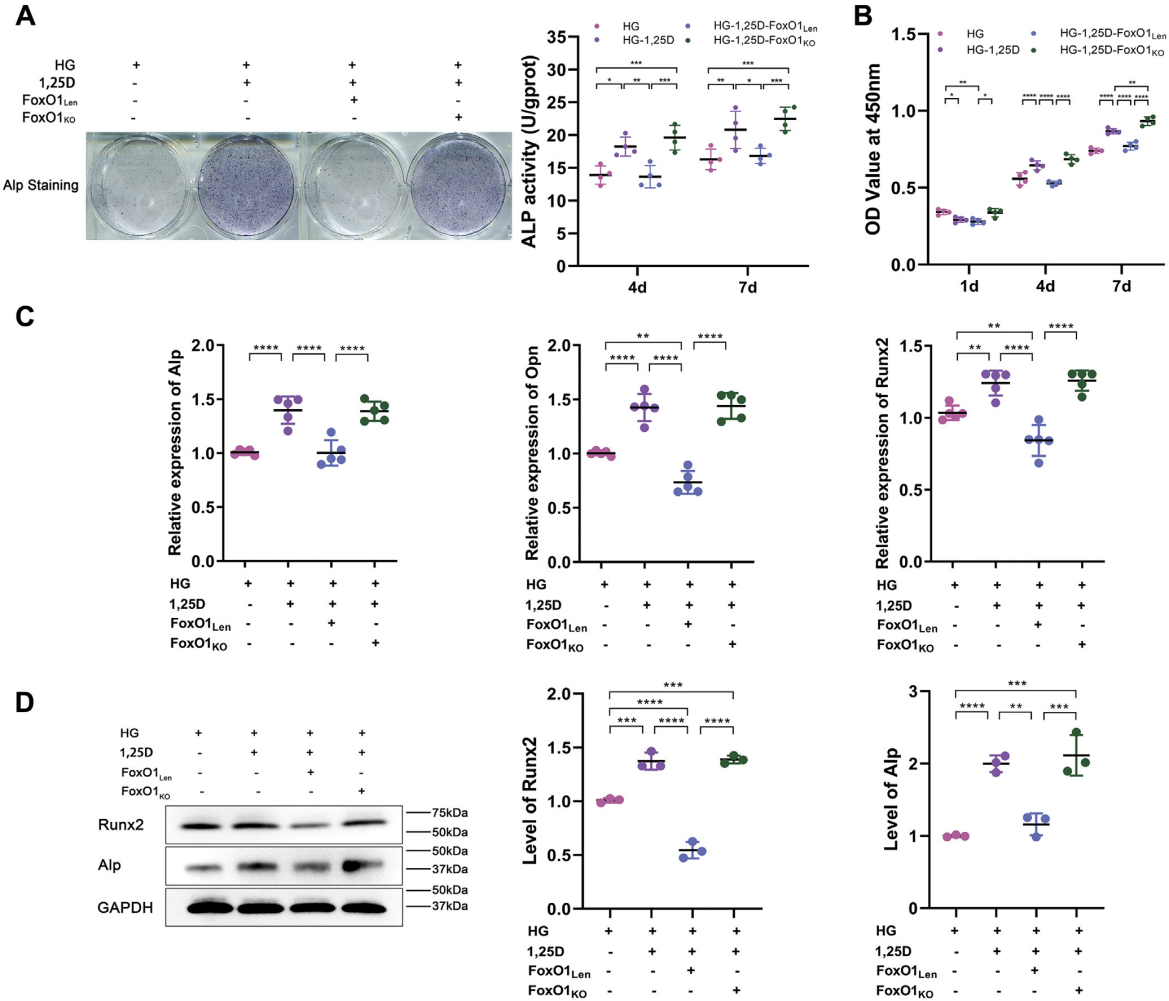
**1,25D treatment and FoxO1 knockout promotes biological functions of osteoblasts under high-glucose condition.***A*, differentiation of osteoblasts was detected by alkaline phosphatase (ALP) activity at 4 days and 7 days and Alp staining after osteogenic induction in different groups, n = 4. *B*, cell viability determined by CCK-8 assay at 1 day, 4 days, and 7 days in different groups, n = 4. *C*, the mRNA expression levels of alkaline phosphatase (Alp), osteopontin (Opn), and runt-related transcription factor 2 (Runx2) were tested by real-time qPCR analysis, n = 5. *D*, protein expression levels of Alp and Runx2 were evaluated using western blot analysis, n = 3. Data are expressed as mean ± standard deviation (SD) obtained in three independent experiments, ns represents *p* > 0.05, ∗*p* < 0.05, ∗∗*p* < 0.01, ∗∗∗*p* < 0.001, ∗∗∗∗*p* < 0.0001. FoxO1_KO_, conditional knockout of FoxO1; FoxO1_Len_, lentivirus-mediated overexpression of FoxO1; HG, high glucose.

In contrast to HG-1,25D group, ALP activity decreased notably in HG-1,25D-FoxO1_Len_ group, while it was potentiated in HG-1,25D-FoxO1_KO_ group (Fig. 8*A*). According to CCK-8 assay, cell proliferation was promoted by 1,25D under HG condition. But it was impaired when FoxO1 overexpressed, which abrogated the positive influence of 1,25D. However, FoxO1 knockout enhanced the proliferative capacity of primary osteoblasts (Fig. 8*B*). Through real-time qPCR, we observed that 1,25D promoted the mRNA expressions of Alp, Opn, and Runx2 in HG-treated primary osteoblasts, but FoxO1 overexpression resulted in a decreased mRNA level of these genes. Conversely, these markers were upregulated when FoxO1 was knocked out (Fig. 8*C*). Also, protein expressions of Alp and Runx2 showed similar variations (Fig. 8*D*), indicating that FoxO1 was an important mediator in the regulation of autophagy in HG-treated primary osteoblasts by 1,25D. Taken together, these data manifested that 1,25D could improve biological functions of primary osteoblasts by attenuating HG-induced excessive autophagy, and FoxO1 inhibition played a critical role in this process.

## Discussion

Bone metabolism abnormality is one of the complications in diabetes (3). Positive effects of vitamin D on diabetes-induced osteopenia have become a hot topic in this realm. In our *in vivo* study, we confirmed that 1,25D rescued glucose metabolism disorder and bone microstructure deterioration in diabetic mice. *In vitro*, 1,25D improved osteoblasts viability and differentiation in HG environment. The oxidative stress caused by diabetes has adverse effects on bone formation, during which the accumulation of ROS would increase (36). But 1,25D downregulated ROS level, implying that it might exert osteoprotective effects in the progression of DM through ameliorating redox imbalance.

Increasing evidence has suggested that autophagy is involved in metabolic dysfunction in the DM development (37, 38). Consistent with previous research demonstrating that autophagy was triggered in MC3T3-E1 cells by HG (39), we found that autophagy presented time-dependent accumulation in osteoblasts cultured in HG, as visualized by upregulated level of Beclin1 and LC3II/LC3I ratio. The longer HG treatment it took, the higher level of autophagic activity was observed in osteoblasts, indicating that long-term hyperglycemia led to excessive autophagy. Additionally, western blot analysis verified that 1,25D could alleviate elevated autophagy caused by HG in osteoblasts, and its effect was dose-dependent. Based on the above, our findings suggested that enhanced autophagy could be deleterious to osteoblasts under diabetic condition, and 1,25D might improve diabetes-mediated metabolism abnormality by attenuating autophagy.

However, as an essential mechanism sustaining cellular homeostasis, basal rates of autophagy are considered to play a protective role in osteoblastic cells cultured in HG medium (40). And deficiency of autophagy could impede osteoblasts differentiation and mineralization (41). These findings have shed light on the positive role of autophagy induction in the maintenance of bone homeostasis. Nevertheless, elevation of autophagy might not always exert favorable effects, and excessive autophagy could be detrimental to cells (42, 43). In an attempt to have a better knowledge about the effects of autophagy, we used autophagy activator RAP. Our data revealed that 1,25D treatment led to a sharply decreased autophagic flux in HG-treated osteoblasts, but RAP notably increased the number of autolysosomes and autophagosomes in cells. Simultaneously, we observed that differentiative capacity of osteoblasts was suppressed obviously following RAP application after 5 days. Also, CCK-8 assay displayed that the proliferation of osteoblasts reduced significantly after 4-day intervention. These results demonstrated that RAP-induced excessive autophagy ablated positive impacts of 1,25D on osteoblasts differentiation and proliferation. Of note, we found that the proliferative capacity of osteoblasts slightly increased with RAP addition at 1 day, indicating that autophagy might have biphasic effects according to the time of intervention. In other words, enhanced autophagic activity in HG environment exerted short-term osteoprotective effects, but excessive autophagy under long-term hyperglycemia could have inhibitory effects on the biological properties of osteoblastic cells. Therefore, autophagic activity should be controlled at a proper level. Given that diabetes is a long-term chronic disease, the short-term HG models *in vitro* in many studies might not exactly simulate the natural developing environment in diabetes. Moreover, the concentration of glucose and the duration of exposure are diverse in different models, which could also be responsible for those controversial results.

It has been considered that insulin could regulate cell metabolism through PI3K signal pathway. But other growth factors present in serum such as cytokines might also be involved in this process. Previous studies have explored the role of insulin using osteoblast-like cells incubated in the absence of serum and confirmed that insulin was responsible for stimulating PI3K/Akt signaling cascade (44, 45). Thus, elevated serum insulin level mediated by 1,25D *in vivo* study might be an explanation for the activation of PI3K/Akt signaling by 1,25D in hyperglycemia environment. After PI3K/Akt activation, we observed that FoxO1 was phosphorylated and its nuclear exclusion was enhanced. Phosphorylation of FoxO1 is an essential event that determines its transcriptional activity and intracellular location (26, 46). Dephosphorylated FoxO1 has high transcriptional capacity located in the nucleus (47). 1,25D augmented p-FoxO1 production, which had low transcriptional activity and promoted the shuttle of FoxO1 from nucleus to cytoplasm. That is, 1,25D stimulated PI3K/Akt signaling and inhibited its downstream target FoxO1. When FoxO1 was restrained, Sestrin3 (Sesn3)/AMPK signaling transduction pathway was blocked, which could activate mammalian target of rapamycin complex 1 (mTORC1) (48). Since mTORC1 is a major negative regulator of autophagy participating in the modulation of lysosomal biogenesis (49), FoxO1-mediated mTORC1 suppression could activate autophagy. In the present study, FoxO1 was inhibited by 1,25D through PI3K/Akt signaling. The inactivated FoxO1-mediated-mTORC1 activation could lead to autophagy inhibition, which was beneficial to osteogenic facilitation in HG environment. Nevertheless, RAP regulates autophagy by directly inhibiting mTORC1 (50), which might account for the results that 1,25D couldn’t mitigate elevated autophagy and subsequent negative effects on osteogenesis induced by RAP. In addition, we found that ROS scavenger could reverse HG-induced FoxO1 activation, indicating that ROS could play a critical role in FoxO1 distribution and activity in the diabetic environment. Taken together, these findings suggested that 1,25D might affect FoxO1 signaling through suppressing oxidative stress, thereby leading to osteoprotective process.

It should be noted that the role of FoxO1 varies in different environment. The existence of FoxO1 is a pivotal factor contributing to the bone formation under normal conditions, while its activation aggravates impaired osteogenesis under hyperglycemia (21, 51). In this study reported here, we found that overexpression of FoxO1 resulted in an increased level of autophagy, thus blocking the osteoprotective function of 1,25D. While FoxO1 deficiency indicated synergistic effects with 1,25D treatment, both of which alleviated intracellular autophagy and then improved biological functions of osteoblasts in HG environment. Based on the above, we illuminated that 1,25D could ameliorate diabetes-induced bone loss by attenuating autophagy, and PI3K/Akt/FoxO1 signaling pathway might be one of the mechanisms involved.

However, there were still some limitations in this work. One caveat was the animal model. Low-dose STZ-induced diabetic mouse models might be more useful for characterizing type 1 diabetes mellitus. The treatment of high-fat feeding supplemented with low-to-moderate doses of STZ might more resemble to human type 2 diabetes mellitus (T2DM). But most T2DM patients display damaged not only insulin production, but also insulin resistance (52). Since these models couldn’t really mimic either type 1 diabetes mellitus or T2DM of this condition in human, the animal model in the present study was just a model of chronic hyperglycemia. Additionally, experiments were all performed in mouse tissues. In *in vitro* studies, osteoblasts cultured in pure HG couldn’t completely mimic the *in vivo* diabetic microenvironment. For these reasons, it should be cautious for extrapolation of the findings directly to human, and further studies need to be investigated. Taking these aspects into consideration, this study still provided a significant, if not sufficient, contribution to understand promotive role of 1,25D in diabetic bone metabolism.

To summarize, our results clearly elucidated that 1,25D could reverse dysfunctional bone metabolism through attenuating autophagy. In this process, PI3K/Akt/FoxO1 signaling transduction pathway played a pivotal role. These findings also highlighted the significance of autophagy in diabetes, which should be controlled in an appropriate degree. Our study could provide a therapeutic strategy for the treatment of diabetes-related bone diseases.

## Experimental procedures

### Animal study

FVB.129S6(Cg)-FoxO1<tm1Rdp>/J (FoxO1^fl/fl^) mice and B6N.FVB-Tg (BGLAP-cre) 1Clem/J (Cre^+/−^) mice were purchased from Jackson Laboratories. FoxO1^fl/−^Cre^+/−^ mice were obtained by crossing FoxO1^fl/fl^ mice and Cre^+/−^ mice. Subsequently, FoxO1^fl/−^Cre^+/−^ mice were crossed with FoxO1^fl/fl^ mice. Then FoxO1_OB_^−/−^ mice (KO) and their WT littermates were obtained (35). Phenotypes of mice were identified using real-time qPCR and western blot (Fig. S1). All animal experiments were conducted in accordance with international standards on animal welfare and approved by the Animal Research Committee of Sichuan University (Chengdu, China).

*In vivo*, we only used WT mice. Twenty-four male WT mice of 4 weeks old were randomly divided into three groups: control, DM, and DM-1,25D. Mice in the latter two groups were on a high-fat diet (60 kcal% fat, 20 kcal% carbohydrate, 20 kcal% protein; D12492 diet; Research Diets) for 3 weeks before streptozotocin (STZ, Sigma) injection. On day 22, mice fasted for 6 to 8 h and then injected STZ at a repeated dose of 40 mg/kg. High-fat food was then provided as before. Ten days after STZ administration, the level of blood glucose was measured from a tail vein blood sample of each mouse. Mice with blood glucose>150 mg/dl higher than control mice were used in this study.

1,25D, dissolved in propylene glycol and alcohol (4:1), was administered intraperitoneally at a dose of 5ug/kg every other day for 4 weeks. Then serum and bone tissues were harvested for following experiments.

### Serum glucose and enzyme-linked immunosorbent assay (ELISA)

Mice were sacrificed and the serum glucose level was examined by the glucose oxidase method. Quantitative examination of serum value of insulin (Mercodia), 1,25D (IdT), 25(OH)D_3_ (R&D System), Ocn (Jiancheng), and Alp (Jiancheng) was performed from the supernatant of each sample *via* ELISA kit following the manufacturer's instructions.

### Micro-CT analysis

Femurs were collected and fixed after 4 weeks of 1,25D treatment, scanning by micro-CT (Micro CT 50, SCANCO Medical AG) at 7 μm resolution. Three-dimensional (3D) images of bones were reconstructed. Also, bone tissues were tested and analyzed by the script of BMD, BV/TV, Tb.N, and Tb.Sp.

### Histology, histomorphometry, and immunohistochemistry

Specimens from distal femurs were collected and maintained in 4% neutral Paraformaldehyde for 2 days. Then they were fixed, decalcified in 10% ethylene diamine tetraacetic acid (EDTA) for 4 weeks, dehydrated through a graded series of ethanol solutions, and embedded in paraffin. These samples were sawn into 5 μm longitudinal sections for HE and TRAP staining. Histomorphometric measurement—a number of osteoclasts were tested using the OsteoMeasure Analysis System (OsteoMetrics, Inc) as previously reported (53). Besides, some sections were immunostained for Runx2 (Abcam) and Opn (Abcam) using respective rabbit monoclonal antibodies.

### Cell culture and construction of FoxO1 overexpression lentiviral vector system

Osteoblasts were isolated from calvaria of newborn WT and KO mice (2 days old), respectively. Calvaria were dissected from these mice aseptically and placed in tissue culture dish containing phosphate buffer saline (PBS) with penicillin and streptomycin. Then the calvaria were cut into 0.5 × 0.5 mm^2^ pieces after stripping off the periosteum and moved into a culture flask containing α-MEM (Gibco) supplemented with 10% fetal bovine serum (FBS, Biowest). The inverted flask was placed in incubator at 37 °C in 5% CO_2_ and turned over 2 h later. Medium was replaced every 3 days, and cells were passaged when they reached 80 to 90% confluence. Those in passage 3 were used in this study.

To establish the FoxO1 overexpression model in osteoblasts, we applied lentivirus-FoxO1 (GENECHEM Co) to transfect osteoblasts for 10 h at an MOI of 40. Cells were treated as follow groups: control, HG, HG and 1,25D (10^−8^ mol/l; Sigma) (HG-1,25D), HG and 1,25D supplemented with 100 nM RAP (Sigma) (HG-1,25D-RAP), HG and 1,25D supplemented with lentivirus-mediated overexpression of FoxO1 (HG-1,25D-FoxO1_Len_), HG and 1,25D treated conditional knockout osteoblasts (HG-1,25D-FoxO1_KO_). The former four groups used osteoblasts isolated from WT mice, while the last group used osteoblasts isolated from KO mice. For the HG groups, the glucose was applied at a concentration of 22 mmol/l.

### Cell biology

Osteoblasts were seeded at a density of 5 × 10^3^ cells per well in 96-well plates in a total of five replicate wells each group. Cell proliferation was tested by Cell Counting Kit-8 (CCK-8, Dojindo) assay at the time point of 1 day, 4 days, and 7 days. The optical density (OD) value at 450 nm was measured by a microplate reader (NanoDrop).

For osteogenic differentiation, osteoblasts were cultured in osteogenic induction medium (α-MEM, 10mML-ascorbic acids, 10% FBS, antibiotics, 10 mM β-glycerophosphate, and 100 nM dexamethasone (Gibco)). At 4 days and 7 days after treatment, cells were collected and ALP activity was estimated by ALP Assay Kit (Beyotime). Total protein levels in the same samples were examined using BCA Protein Assay Kit (Beyotime). The ALP activity was normalized to total protein as nanomoles of produced p-nitrophenol per minute per mg of protein (nmol/min/mg protein). In addition, Alp and alizarin red staining were carried out after 21-day osteogenic induction to further observe the osteogenic differentiation of osteoblasts. Cells were fixed by 4% paraformaldehyde and stained by Alp staining kit (Beyotime) and alizarin red (Sigma) for 30 min. Then the cells were rinsed with PBS (Gibco) for three times and photographed after the plate got dry.

### Intracellular ROS formation

The intracellular ROS level in each group was determined using 2’,7’-dichlorodihydrofluorescin diacetate (DCFH-DA, Molecular Probes, Invitrogen), which was an oxidation sensitive fluorescent probe dye. Osteoblasts were incubated with 10 μM DCFH-DA for 30 min at 37 °C. Then flow cytometry was performed and fluorescence was evaluated by LSR II Flow cytometer (BD biosciences) at an excitation wavelength of 495 nm and emission wavelength of 527 nm. There were three biological replicates in each group.

### Real-time qPCR and western blot

Cells in different groups were collected at the time point of 5 days and total RNAs were extracted by Trizol Reagent (Invitrogen). Reverse transcriptions were conducted using Prime Script Reverse Transcriptase (Takara) and real-time qPCR was carried out by SYBR Premix Ex TaqTM kit (Takara) with ABI 7300 real-time PCR system (Applied Biosystems). The expressions of osteogenic markers such as Alp, Opn, and Runx2 were respectively calculated. Primer sequences were listed in Table 1. Endogenous housekeeping gene glyceraldehyde-3-phosphate dehydrogenase (GAPDH) was used as loading control.

**Table 1:** Primers sequences for real time-qPCR

Gene	Forward 5'→3'	Reverse 5'→3'
Alp	AACCCAGACACAAGCATTCC	GCCTTTGAGGTTTTTGGTCA
Opn	CCCGGTGAAAGTGACTGATT	TTCTTCAGAGGACACAGCATTC
Runx2	GGCGTCAAACAGCCTCTTCA	GCTCACGTCGCTCATCTTGC
GAPDH	AAGGCCGGGGCCCACTTGAA	GGACTGTGGTCATGAGCCCTTCCA

Alp, alkaline phosphatase; GAPDH, glyceraldehyde-3-phosphate dehydrogenase; Opn, osteopontin; Runx2, runt-related transcription factor 2.

For western blot analysis, osteoblasts were scraped in lysis buffer (Keygen total protein extraction kit, Keygen Biotech) in different groups. Protein extracts were separated by 10% SDS-PAGE gels and blotted onto polyvinylidene difluoride membranes (Millipore Corp). After being blocked, the membranes were probed with primary antibodies at 4 °C overnight, which were then incubated with HRP-conjugated secondary antibodies (1:2000) the next day. The bands were exposed and immunoblot images were acquired and measured by Quantity One (Bio-Rad). The primary antibodies used in this study were as follows: Alp (1:500, Abcam), Runx2 (1:500, Abcam), Beclin1 (1:200, Santa Cruz), LC3II (1:1000, Cell Signaling Technology), LC3I (1:1000, Cell Signaling Technology), p-Akt (1:500, Cell Signaling Technology), Akt (1:500, Cell Signaling Technology), p-FoxO1 (1:100, Santa Cruz), FoxO1 (1:100, Santa Cruz), and GAPDH (1:1000, SAB) applied as control for normalization.

### Immunofluorescence and mRFP-GFP-LC3 fluorescence double labeling

Osteoblasts in different groups were fixed by 4% paraformaldehyde for 30 min and then permeabilized by 2% Triton X-100 for 5 min. Bovine serum albumin (Sigma) were added to block cells for 30 min. Next, osteoblasts were incubated with primary antibodies of FoxO1 (1:50, Santa Cruz Biotechnology) at 4 °C overnight. The control group was incubated with PBS. After the subsequent incubation with fluorescein isothiocyanate-conjugated secondary antibodies (1:50, Zsjq Bio Co), nuclei were stained with DAPI (1:1000, Beyotime). Cells were observed using a fluorescent microscopy (Olympus), and quantitation of the distribution of FoxO1 between the nucleus and the cytosol was tested by Image Pro Plus software.

To better understand and detect the autophagic flux in different groups, fluorescence double labeling was performed using mRFP-GFP-LC3 adenovirus (Hanbio Biotechnology Co, Ltd) to trace the formation and degradation of autophagosome in transfected osteoblasts following manufacturer’s instructions. Results were observed through confocal laser scanning microscope (Olympus).

### Statistical analysis

Statistical analysis was performed by SPSS 20.0 software (SPSS, Inc). All quantitative data are presented as mean ± SD with a minimum of three independent samples. Statistical significance was determined by one-way ANOVA followed by Tukey’s test for multiple comparisons and two-way ANOVA followed by Bonferroni’s post hoc test for different groups. A statistically significant difference was assumed at *p* < 0.05 (∗*p* < 0.05, ∗∗*p* < 0.01, ∗∗∗*p* < 0.001, ∗∗∗∗*p* < 0.0001).

## Data availability

All data generated or analyzed in the present study are included in this article or in the data repositories listed in References.

## Conflict of interest

The authors declare that they have no conflict of interest.

## References

[1] Schmidt A.M. (2018). Highlighting diabetes mellitus: The epidemic continues. Arterioscler. Thromb. Vasc. Biol..

[2] Epstein S., Defeudis G., Manfrini S., Napoli N., Pozzilli P. (2016). Diabetes and disordered bone metabolism (diabetic osteodystrophy): Time for recognition. Osteoporos. Int..

[3] Lecka-Czernik B. (2017). Diabetes, bone and glucose-lowering agents: Basic biology. Diabetologia.

[4] Sellmeyer D.E., Civitelli R., Hofbauer L.C., Khosla S., Lecka-Czernik B., Schwartz A.V. (2016). Skeletal metabolism, fracture risk, and fracture outcomes in type 1 and type 2 diabetes. Diabetes.

[5] Dusso A.S. (2011). Kidney disease and vitamin D levels: 25-hydroxyvitamin D, 1,25-dihydroxyvitamin D, and VDR activation. Kidney Int. Suppl..

[6] Welsh J. (2018). Vitamin D and breast cancer: Past and present. J. Steroid Biochem. Mol. Biol..

[7] Mitri J., Pittas A.G. (2014). Vitamin D and diabetes. Endocrinol. Metab. Clin. North Am..

[8] Sacerdote A., Dave P., Lokshin V., Bahtiyar G. (2019). Type 2 diabetes mellitus, insulin resistance, and vitamin D. Curr. Diabetes Rep..

[9] Levine B., Kroemer G. (2008). Autophagy in the pathogenesis of disease. Cell.

[10] Amaravadi R., Kimmelman A.C., White E. (2016). Recent insights into the function of autophagy in cancer. Genes Dev..

[11] Guo F., Liu X., Cai H., Le W. (2018). Autophagy in neurodegenerative diseases: Pathogenesis and therapy. Brain Pathol..

[12] Levine B., Kroemer G. (2019). Biological functions of autophagy genes: A disease perspective. Cell.

[13] Marasco M.R., Linnemann A.K. (2018). β-Cell autophagy in diabetes pathogenesis. Endocrinology.

[14] Lee C.T., Wang J.Y., Chou K.Y., Hsu M.I. (2019). 1,25-Dihydroxyvitamin D(3) modulates the effects of sublethal BPA on mitochondrial function via activating PI3K-Akt pathway and 17β-estradiol secretion in rat granulosa cells. J. Steroid Biochem. Mol. Biol..

[15] Zhang X., Zanello L.P. (2008). Vitamin D receptor-dependent 1 alpha,25(OH)2 vitamin D3-induced anti-apoptotic PI3K/AKT signaling in osteoblasts. J. Bone Miner. Res..

[16] Vanhaesebroeck B., Guillermet-Guibert J., Graupera M., Bilanges B. (2010). The emerging mechanisms of isoform-specific PI3K signalling. Nat. Rev. Mol. Cell Biol..

[17] Huang X., Liu G., Guo J., Su Z. (2018). The PI3K/AKT pathway in obesity and type 2 diabetes. Int. J. Biol. Sci..

[18] Yan J., Wang C., Jin Y., Meng Q., Liu Q., Liu Z., Liu K., Sun H. (2018). Catalpol ameliorates hepatic insulin resistance in type 2 diabetes through acting on AMPK/NOX4/PI3K/AKT pathway. Pharmacol. Res..

[19] Yao H., Yao Z., Zhang S., Zhang W., Zhou W. (2018). Upregulation of SIRT1 inhibits H2O2-induced osteoblast apoptosis via FoxO1/β-catenin pathway. Mol. Med. Rep..

[20] Rached M.T., Kode A., Xu L., Yoshikawa Y., Paik J.H., Depinho R.A., Kousteni S. (2010). FoxO1 is a positive regulator of bone formation by favoring protein synthesis and resistance to oxidative stress in osteoblasts. Cell Metab..

[21] Teixeira C.C., Liu Y., Thant L.M., Pang J., Palmer G., Alikhani M. (2010). Foxo1, a novel regulator of osteoblast differentiation and skeletogenesis. J. Biol. Chem..

[22] Saline M., Badertscher L., Wolter M., Lau R., Gunnarsson A., Jacso T., Norris T., Ottmann C., Snijder A. (2019). AMPK and AKT protein kinases hierarchically phosphorylate the N-terminus of the FOXO1 transcription factor, modulating interactions with 14-3-3 proteins. J. Biol. Chem..

[23] Milan G., Romanello V., Pescatore F., Armani A., Paik J.H., Frasson L., Seydel A., Zhao J., Abraham R., Goldberg A.L., Blaauw B., DePinho R.A., Sandri M. (2015). Regulation of autophagy and the ubiquitin-proteasome system by the FoxO transcriptional network during muscle atrophy. Nat. Commun..

[24] I O.S., Zhang W., Wasserman D.H., Liew C.W., Liu J., Paik J., DePinho R.A., Stolz D.B., Kahn C.R., Schwartz M.W., Unterman T.G. (2015). FoxO1 integrates direct and indirect effects of insulin on hepatic glucose production and glucose utilization. Nat. Commun..

[25] Lu M., Wan M., Leavens K.F., Chu Q., Monks B.R., Fernandez S., Ahima R.S., Ueki K., Kahn C.R., Birnbaum M.J. (2012). Insulin regulates liver metabolism *in vivo* in the absence of hepatic Akt and Foxo1. Nat. Med..

[26] Guo X., Lin H., Liu J., Wang D., Li D., Jiang C., Tang Y., Wang J., Zhang T., Li Y., Yao P. (2020). 1,25-Dihydroxyvitamin D attenuates diabetic cardiac autophagy and damage by vitamin D receptor-mediated suppression of FoxO1 translocation. J. Nutr. Biochem..

[27] Liu L., Zheng L.D., Zou P., Brooke J., Smith C., Long Y.C., Almeida F.A., Liu D., Cheng Z. (2016). FoxO1 antagonist suppresses autophagy and lipid droplet growth in adipocytes. Cell Cycle.

[28] Matsuzaki T., Alvarez-Garcia O., Mokuda S., Nagira K., Olmer M., Gamini R., Miyata K., Akasaki Y., Su A.I., Asahara H., Lotz M.K. (2018). FoxO transcription factors modulate autophagy and proteoglycan 4 in cartilage homeostasis and osteoarthritis. Sci. Transl. Med..

[29] Petrocca F., Visone R., Onelli M.R., Shah M.H., Nicoloso M.S., de Martino I., Iliopoulos D., Pilozzi E., Liu C.G., Negrini M., Cavazzini L., Volinia S., Alder H., Ruco L.P., Baldassarre G. (2008). E2F1-regulated microRNAs impair TGFbeta-dependent cell-cycle arrest and apoptosis in gastric cancer. Cancer Cell.

[30] Cao Y., Klionsky D.J. (2007). Physiological functions of Atg6/Beclin 1: A unique autophagy-related protein. Cell Res..

[31] Levine B., Liu R., Dong X., Zhong Q. (2015). Beclin orthologs: Integrative hubs of cell signaling, membrane trafficking, and physiology. Trends Cell Biol..

[32] Mizushima N., Yamamoto A., Matsui M., Yoshimori T., Ohsumi Y. (2004). *In vivo* analysis of autophagy in response to nutrient starvation using transgenic mice expressing a fluorescent autophagosome marker. Mol. Biol. Cell.

[33] Mizushima N., Yoshimori T., Levine B. (2010). Methods in mammalian autophagy research. Cell.

[34] Wymann M.P., Zvelebil M., Laffargue M. (2003). Phosphoinositide 3-kinase signalling--which way to target?. Trends Pharmacol. Sci..

[35] Xiong Y., Zhang Y., Xin N., Yuan Y., Zhang Q., Gong P., Wu Y. (2017). 1alpha,25-Dihydroxyvitamin D3 promotes bone formation by promoting nuclear exclusion of the FoxO1 transcription factor in diabetic mice. J. Biol. Chem..

[36] Napoli N., Chandran M., Pierroz D.D., Abrahamsen B., Schwartz A.V., Ferrari S.L. (2017). Mechanisms of diabetes mellitus-induced bone fragility. Nat. Rev. Endocrinol..

[37] Kim K.H., Lee M.S. (2014). Autophagy--a key player in cellular and body metabolism. Nat. Rev. Endocrinol..

[38] Kim J., Lim Y.M., Lee M.S. (2018). The role of autophagy in systemic metabolism and human-type diabetes. Mol. Cell.

[39] Wang X., Feng Z., Li J., Chen L., Tang W. (2016). High glucose induces autophagy of MC3T3-E1 cells via ROS-AKT-mTOR axis. Mol. Cell. Endocrinol..

[40] Bartolomé A., López-Herradón A., Portal-Núñez S., García-Aguilar A., Esbrit P., Benito M., Guillén C. (2013). Autophagy impairment aggravates the inhibitory effects of high glucose on osteoblast viability and function. Biochem. J..

[41] Li H., Li D., Ma Z., Qian Z., Kang X., Jin X., Li F., Wang X., Chen Q., Sun H., Wu S. (2018). Defective autophagy in osteoblasts induces endoplasmic reticulum stress and causes remarkable bone loss. Autophagy.

[42] Yamamoto S., Kazama J.J., Fukagawa M. (2013). Autophagy: A two-edged sword in diabetes mellitus. Biochem. J..

[43] Meng H.Z., Zhang W.L., Liu F., Yang M.W. (2015). Advanced glycation end products affect osteoblast proliferation and function by modulating autophagy via the receptor of advanced glycation end products/Raf protein/mitogen-activated protein kinase/extracellular signal-regulated kinase kinase/extracellular signal-regulated kinase (RAGE/Raf/MEK/ERK) pathway. J. Biol. Chem..

[44] Ramalingam M., Kwon Y.D., Kim S.J. (2016). Insulin as a potent stimulator of Akt, ERK and inhibin-βE signaling in osteoblast-like UMR-106 cells. Biomol. Ther..

[45] Yang J., Zhang X., Wang W., Liu J. (2010). Insulin stimulates osteoblast proliferation and differentiation through ERK and PI3K in MG-63 cells. Cell Biochem. Funct..

[46] van der Horst A., Burgering B.M. (2007). Stressing the role of FoxO proteins in lifespan and disease. Nat. Rev. Mol. Cell Biol..

[47] Cao J., Yu Y., Zhang Z., Chen X., Hu Z., Tong Q., Chang J., Feng X.H., Lin X. (2018). SCP4 promotes gluconeogenesis through FoxO1/3a dephosphorylation. Diabetes.

[48] Chen C.C., Jeon S.M., Bhaskar P.T., Nogueira V., Sundararajan D., Tonic I., Park Y., Hay N. (2010). FoxOs inhibit mTORC1 and activate Akt by inducing the expression of Sestrin3 and Rictor. Dev. Cell.

[49] Hosokawa N., Hara T., Kaizuka T., Kishi C., Takamura A., Miura Y., Iemura S., Natsume T., Takehana K., Yamada N., Guan J.L., Oshiro N., Mizushima N. (2009). Nutrient-dependent mTORC1 association with the ULK1-Atg13-FIP200 complex required for autophagy. Mol. Biol. Cell.

[50] Schreiber K.H., Arriola Apelo S.I., Yu D., Brinkman J.A., Velarde M.C., Syed F.A., Liao C.Y., Baar E.L., Carbajal K.A., Sherman D.S., Ortiz D., Brunauer R., Yang S.E., Tzannis S.T., Kennedy B.K. (2019). A novel rapamycin analog is highly selective for mTORC1 *in vivo*. Nat. Commun..

[51] Iyer S., Han L., Ambrogini E., Yavropoulou M., Fowlkes J., Manolagas S.C., Almeida M. (2017). Deletion of FoxO1, 3, and 4 in osteoblast progenitors attenuates the loss of cancellous bone mass in a mouse model of type 1 diabetes. J. Bone Miner. Res..

[52] Furman B.L. (2015). Streptozotocin-induced diabetic models in mice and rats. Curr. Protoc. Pharmacol..

[53] Dempster D.W., Compston J.E., Drezner M.K., Glorieux F.H., Kanis J.A., Malluche H., Meunier P.J., Ott S.M., Recker R.R., Parfitt A.M. (2013). Standardized nomenclature, symbols, and units for bone histomorphometry: A 2012 update of the report of the ASBMR Histomorphometry Nomenclature Committee. J. Bone Miner. Res..

