# Supplementary material for: 1α,25-Dihydroxyvitamin D3 ameliorates diabetes-induced bone loss by attenuating FoxO1-mediated autophagy
Source: J Biol Chem. 2021 Jan 13;296:100287. doi: 10.1016/j.jbc.2021.100287 (PMC7948959; doi:10.1016/j.jbc.2021.100287)
Supplement: Supplemental Figure S1 and Tables S1–S2 [file mmc1.pdf]

1α,25-Dihydroxyvitamin D3 ameliorates diabetes-induced bone loss by attenuating FoxO1-mediated autophagy

Yixuan Jiang[1,2], Wenqiong Luo[1,2], Bin Wang[1,2], Zumu Yi[1], Ping Gong[1,2], Yi Xiong[1,2,*]

[1] State Key Laboratory of Oral Diseases, National Clinical Research Center for Oral Diseases, West China Hospital of Stomatology, Sichuan University, Chengdu, China
[2] Department of Oral Implantology, West China Hospital of Stomatology, Sichuan University, Chengdu, China

* Corresponding author: Yi Xiong
E-mail: xiongraise@163.com (YX)

Running title: 1,25D prevents bone loss via FoxO1-mediated autophagy

Keywords: 1α,25-Dihydroxyvitamin D3 (1,25D), autophagy, forkhead transcription factor 1 (FoxO1), osteogenesis, diabetes mellitus

Table S1 Detection of bone trabecular parameters in different groups.

Group	BMD (mg HA/cm^3)	BV/TV	Tb.N (/mm)	Tb.Sp (mm)
Ctrl	796.4±16.35	0.59±0.11	7.60±0.99	0.36±0.07
Ctrl-1,25D	808.6±25.87	0.60±0.05	7.20±0.60	0.40±0.03
DM	708.3±10.04$^{*\#}$	0.21±0.06$^{*\#}$	3.16±0.46$^{*\#}$	0.84±0.13$^{*\#}$
DM-1,25D	746.2±15.07$^{*\#\$}$	0.37±0.06$^{*\#\$}$	4.64±1.01$^{*\#\$}$	0.66±0.06$^{*\#\$}$

$^{*}p<0.05$，compared with Ctrl；$^{\#}p<0.05$，compared with Ctrl-1,25D；$^{\$}p<0.05$，compared with DM. Ctrl: control; DM: diabetes mellitus; BMD: bone mineral density; BV/TV: bone volume per total volume; Tb.N: mean trabecular number; Tb.Sp: trabecular separation.

Table S2 Serological tests in different groups.

Group	Serum Alp (U/L)	Serum Ocn (ng/ml)	Serum glucose (mg/dl)
Ctrl	11.8±2.77	273.3±19.8	165.9±14.4
Ctrl-1,25D	12.3±1.56	295.0±14.8	142.9±18.4
DM	4.58±1.47$^{*\#}$	96.8±19.3$^{*\#}$	343.1±20.9$^{*\#}$
DM-1,25D	8.04±1.23$^{*\#\$}$	185±12.9$^{*\#\$}$	263.6±26.7$^{*\#\$}$

$^{*}p<0.05$，compared with Ctrl；$^{\#}p<0.05$，compared with Ctrl-1,25D；$^{\$}p<0.05$，compared with DM. Ctrl: control; DM: diabetes mellitus; Alp: alkaline phosphatase; Ocn: osteocalcin.

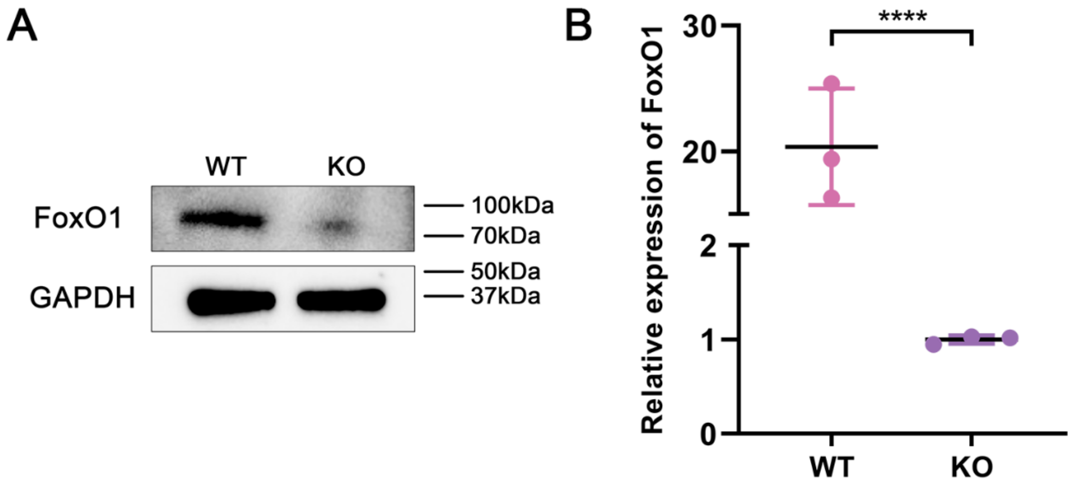

Figure S1. Evidence of FoxO1 deletion *in vivo*.

(A) Protein expression level of FoxO1 examined by Western blot. (B) The mRNA expression of FoxO1 tested using real time-qPCR. WT: wild type mice; KO: $FoxO1_{OB}^{-/-}$ mice.
